# Long-Term Risk of Progression to Sustained Hypertension in White-Coat Hypertension with Normal Night-Time Blood Pressure Values

**DOI:** 10.1155/2020/8817544

**Published:** 2020-12-22

**Authors:** João Faria, José Mesquita Bastos, Susana Bertoquini, José Silva, Jorge Polónia

**Affiliations:** ^1^Department Medicine and Cintesis, Faculty of Medicine, University of Porto, Porto, Portugal; ^2^Health School of Aveiro University, Aveiro, Portugal; ^3^Hypertension Unit, Hospital Pedro Hispano, ULS, Matosinhos, Portugal

## Abstract

**Background:**

The long-term prognosis and transition towards sustained ambulatory hypertension (SHT) of white-coat hypertension (WCHT) remain uncertain particularly in those with both normal nighttime and daytime blood pressure (BP) values. Different classification criteria and the use of antihypertensive drugs may contribute to conflicting results. *Patients and Methods.* We prospectively evaluated for a 7.1 year transition to SHT in 899 nondiabetic subjects free from cardiovascular (CV) events: normotensive (NT) (*n* = 344; 52, 9% female; ageing 48 ± 14 years); untreated WCHT (UnWCHT *n* = 399; 50, 1% female; ageing 51 ± 14 years); and treated WCHT with antihypertensive drugs after baseline (TxWCHT *n* = 156; 54, 4% female; ageing 51 ± 15 years). All underwent 24 h ambulatory BP monitoring (24 h-ABPM) at baseline, at 30 to 60 months, and at 70 to 120 months thereafter. WCHT was at baseline (with no treatment) as office BP ≥ 140/or 90 mm·Hg, daytime BP < 135/85 mm·Hg, and nighttime BP < 120/70 mm·Hg. Development of SHT was considered if daytime BP ≥ 135/or 85 mm Hg and/or nighttime BP ≥ 120/or 70 mm·Hg.

**Results:**

Baseline metabolic parameters did not differ among groups. At 30–60 months and at the end of follow-up, development of SHT occurred, respectively, in NT (3.8% (*n* = 13) and 9.6% (*n* = 33)) and in UnWCHT (10.1% (*n* = 40) and 16.5% (*n* = 66)) (*p* < 0.009). The mean annual increase of average 24 h-systolic BP was 0.48 + 0.93 in NT and 0.73 + 1.06 in UnWCHT, whereas annual SBP in office increased in NT by 1.2 + 0.95 but decreased in UnWCHT by 1.36 + 1.35 mm Hg (*p* < 0.01).

**Conclusion:**

Untreated WCHT patients exhibit a faster and a higher risk of developing SHT compared to NT with TxWCHT assuming an intermediate position between them.

## 1. Introduction

White-coat hypertension (WCHT) refers to the untreated condition in which high blood pressure (BP) values in the office coexist with normal BP values when measured by 24 h ambulatory blood pressure monitoring (24-ABPM), home blood pressure monitoring (HBPM), or both [1]. From its recognition in 1987, until now, the clinical prognosis of WCHT remains uncertain. Early longitudinal studies suggested that WCHT would be an innocent entity proposing that WCHT carries a similar risk to normotension and significantly lower than sustained hypertension (SHT) [2–6] even after adjusting for age and cardiovascular (CV) risk factors [4, 7–11]. In contrast, other studies and meta-analyses suggest that WCHT carries a higher risk of CV morbidity and mortality and place it as an intermediate risk entity between NT and SHT [8, 10, 12–19]. However, overall these studies had a short duration and disparate inclusion criteria, particularly with regard to the definition of WCHT itself. In addition, in some studies, WCHT has been associated with a greater risk of transition for SHT, as well as overall increased risk of CV events [20–22]. Such contradictory results may be explained by the heterogeneity of the studied populations, definition of WCHT and inclusion of treated patients. In several studies, classification of WCHT has been based only on daytime BP values but not on nighttime BP (NBP) which is recognized to be a stronger predictor of cardiovascular risk [3, 23–25]. Moreover, whether or not patients with WCHT should receive antihypertensive drugs is also unresolved [1]. In the present study, our goal was to determine the long-term risk of individuals with WCHT with both normal daytime and NBP for progressing to SHT and the impact on it by the antihypertensive medication.

## 2. Methods

### 2.1. Patients and Protocols

We carried out a longitudinal observational study in a group of 989 outpatients selected at a hospital consultation from 3 hospitals in northern Portugal between 1991 and 2008. All subjects with BP > 140/or 90 mm·Hg in office were referred by GPs to the Hospital Blood Pressure Units to perform 24 h ambulatory BP monitoring in order to confirm the diagnosis of sustained hypertension or of WCHT. The population of the present study was recruited from a cohort of 812 normotensives and 617 subjects with WCHT who were followed up for 7.9 years as previously described [8]. The patients were referred to the hospital consultation from the primary health care to perform a 24 h ambulatory blood pressure monitoring (24 h-ABPM). A pool of normotensive subjects was also integrated into the study as previously described [8]. The protocol was carried out according to the WMA Declaration of Helsinki Ethical Principles for Medical Research Involving Human Subjects, and all data collection was approved by the local Hospital Ethical Committee. As in our previous study [8] to be included in the present one, patients had to be aged between 18 and 70 years and they should (i) have no history or clinical evidence of congestive heart failure, cerebrovascular disease, myocardial infarction, coronary bypass or angioplasty, cardiac valve disease, renal insufficiency, peripheral artery disease, atrial fibrillation or other major arrhythmias, or severe hepatic disease, (ii) have no clinical or analytical evidence of diabetes mellitus and of estimated glomerular filtration rate below 60 ml/min/1.73m^2^ (MDRD), and (iii) have neither suspicion of secondary hypertension nor clinical suspicion of sleep apnoea. WCHT was defined at baseline as follows: to be untreated at that time; with office BP of at least 140 or 90 mm·Hg in three previous consecutive recordings and also in the first measurement of the 24 h-ABPM and with mean 24 h-ABPM less than 130/80 mm·Hg, daytime BP less than 135/85 mm·Hg, and NBP less than 120/70 mm·Hg. Thus subjects included in the present analysis who were diagnosed as WCHT were all absolutely untreated at baseline. After this initial selection, some WCHT were subsequently subjected to antihypertensive treatment (now designed as WCHT treated-TxWCHT) at the initiative of the respective GPs, while others remained untreated during the follow-up (now designed as WCHT untreated-UnWCHT). In the present study, we only included subjects from the previous cohort [8] that could be also revaluated with 24 h-ABPM between 30 and 60 months and between 70 and 120 months after the first 24 h-ABPM at baseline. Subjects with WCHT who received prescriptions with any antihypertensive medications in any phase of the follow-up after the baseline (treated WCHT–TxWCHT) were evaluated separately from subjects with WCHT who were free of medication during the follow-up (untreated WCHT-UnWCHT). The decision to treat came from the patient´s medical assistants, and the collection of information regarding previous antihypertensive medication (if any) was carried out at the moments of the two 24 h ambulatory BP evaluations during the follow-up.

### 2.2. Office and Ambulatory Blood Pressure Monitoring

Office BP was evaluated at admission in three different appointments. Brachial BP and heart rate were evaluated in the nondominant arm using an automated digital oscillometric sphygmomanometer (Omron, Model M6; Omron Corporation, Kyoto, Japan). Three readings, 2 min apart, were taken (on the same day) before the installation of the 24 h-ABPM, and the mean of the last two was considered the brachial BP. Twenty-four-hour ABPM was performed during a working day at the entry of the study with SpaceLabs 90207 (SpaceLabs Inc., Redmond, Washington, USA) as described earlier [8, 9, 26]. The monitor was mounted on the nondominant arm between 08 : 00 and 09 : 00 h and was removed 24 h later. The patients were instructed to perform their usual daily activities and were asked to go to bed precisely at 23 : 00 h, to remain in bed until 07 : 00 h, and advised the use of an alarm clock to help wake up. This was confirmed on the basis of the information obtained from the patient's diary. BP was recorded every 20 min during the day (between 07 : 00 and 23 : 00 h) and every 30 min at night (between 23 : 30 and 06 : 30 h). The nocturnal SBP fall (%) was calculated as 100 x (1 − sleep SBP/awake SBP ratio), and the nocturnal DBP fall (%) was calculated as 100 x (1 − sleep DBP/awake DBP ratio). These periods were considered to be representative of the awake and nighttime resting BPs, respectively.

### 2.3. Follow-Up and End Point Evaluation

The patient's medical records were reviewed for the use of antihypertensive drug therapy and further transition to SHT at the time of the two ABPM recordings during the follow-up which occurred from January 1991 to December 2013. All subjects had beginning normal values of both daytime and nighttime BP. The endpoint was the transition to SHT, defined during the follow-up by the presence at any of the two moments of evaluation of a 24-ABPM, a daytime BP ≥ 135/85 mm·Hg and/or NBP ≥ 120/70 mm·Hg either in the populations of normotensives, WCTH untreated, and WCHT treated. As in the previous study [8] the presence/absence of CV events was confirmed by the examination of the patient's medical records until the end of the follow-up period. Cardiovascular events were diagnosed either by the physician who cared for the patients at the time of the events or if death had occurred; information on its cause was obtained either from the patient's physician or otherwise by examination of the official death certificate. In all cases, the diagnosis of CV events was confirmed objectively by an external expert who examined the patient's records and diagnostic procedures. Both fatal and nonfatal cardiovascular events considered consisted of congestive heart failure, cerebrovascular disease, myocardial infarction, angina pectoris, coronary bypass, or angioplasty. Coronary events included sudden death and fatal and nonfatal myocardial infarction or angina pectoris confirmed in the hospital and coronary bypass or angioplasty. Transient ischaemic attack was not considered an event.

### 2.4. Statistical Analysis

Statistical analysis was carried out using the SPSS software (version 13.0; SPSS Inc., Chicago, Illinois, USA). Values of continuous variables are presented as the mean ± SD categorized data as percentages, and differences between the groups were evaluated by one-way analysis of variance. Continuous variables were compared using one-way ANOVA with Tukey Kramer post hoc test. Nonnormal distribution data were assessed by Kruskal–Wallis nonparametric test. Chi-square test was used for group comparisons for categorized data. Statistical significance was considered for a *p* value less than 0.05.

## 3. Results

### 3.1. Study Population

We evaluated at baseline and at the two moments of the follow-up a total of 899 subjects who were 344 normotensives and 555 subjects with WCHT including 399 untreated and 156 treated with antihypertensive drugs. They were selected from a population studied in a previous cohort [8] but among these, we analysed only those who had available and absolutely reliable data of an evaluation with 24 h-ABPM between 30 and 60 months and between 70 and 120 months after the first 24 h-ABPM at baseline. Thus, from the previous cohort [8] and under those criteria, we analysed 344 out of 812 normotensives and 555 out of 617 subjects with WCHT, i.e., including only those who had available two additional ABPM recordings during the follow-up. Out of the 156 treated subjects with WCHT, 48 subjects received treatment before the 30^th^ month of follow-up, and the remaining 108 subjects thereafter until the end of the follow-up (see Section 3.2).

### 3.2. Flow Chart

The median follow-up period was 7.1 + 1.9 years (0.9–10.1). Treatment consisted on: angiotensin receptor blockers (ARBs) single dose (*n* = 54), ARB combined with hydrochlorothiazide, (*n* = 6), angiotensin-converting enzyme inhibitors (ACEin) single dose (*n* = 22), ACEin combined with diuretics (*n* = 27), calcium antagonists single dose (*n* = 20); beta-blockers single dose, (*n* = 19) and Indapamide single dose (*n* = 8).  Table 1 represents the clinical data of the three populations. As shown, subjects with WCHT were slightly older than normotensives and treated WCHT had a higher serum uric acid than the other groups. For the other clinical variables, groups did not differ significantly.  Table 2 shows for the three groups the blood pressure values in office and with 24* *h ABPM both at baseline and at the end of the follow-up as well as the differences of BP calculated between these moments. At baseline, both groups of WCHT had similar BP values, whereas normotensives had lower office SBP and DBP, 24 h SBP and DBP, daytime SBP, NBP, and lower nighttime fall of SBP than both the WCHT groups. At the end of follow-up, both normotensives and treated WCHT had lower SBP and DBP at the office, 24 h, daytime and nighttime than untreated WCHT. At the end of the follow-up, ambulatory BP values of normotensives and treated WCHT were not different but office BP was lower in normotensives. At baseline, the standard deviation (SD) of both 24 h SBP and DBP, as an estimation of BP variability, was significantly lower in the normotensive group (SD of 24 SBP 11.9 + 2.8 and SD 24* *h DBP 10.1 + 2.2) as compared with the WCHT group (SD of 24* *h SBP 14.4 + 3.2 and SD 24* *h DBP 10.9 + 3.6) (*p* < 0.01) for both, respectively.

**Figure 1:**
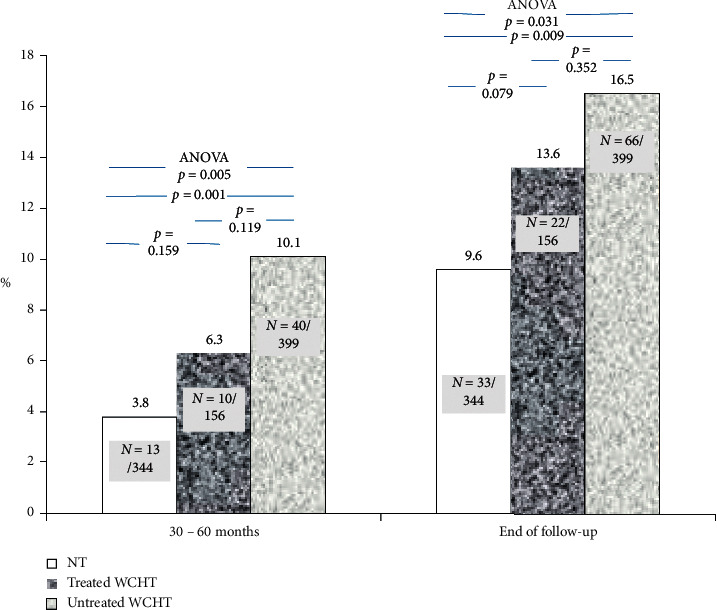
Cumulative incidence and rate (%) of progression to sustained hypertension (SHT) of normotensive subjects (NT) and treated and untreated WCHT subjects. In untreated WCHT, SHT occurred more frequently and earlier than NT.

Concerning the differences (delta) of BP values between baseline and the end of the follow-up, Table 2 shows that office BP increased in normotensives but decreased in subjects with WCHT particularly in the subgroup of those who were treated. From baseline to the end of follow-up, the net increases of 24 h; daytime and nighttime SBP values were significantly lower in treated WCHT than in untreated WCHT.  Figure 1 shows the percentage of subjects who progressed to sustained hypertension.

Both at the end and between 30–60 months of the follow-up, the percentage of normotensives that progressed to SHT was significantly lower than that of subjects with untreated WCHT. Regarding the totality of individuals who at the end of the follow-up progressed to sustained hypertension, in the first assessment at 30–60 months of the follow-up such a progression occurred only in 40% of normotensives, whereas it already occurred in 61% of the untreated WCHT. In other words, in the first assessment at 30–60 months of the follow-up, the percentage of subjects who showed a transition to SHT of untreated subjects with WCHT was 2.7 times higher than normotensives. At the end of the follow-up, these values were 1.7 times vs. that of normotensives. Among subjects who evolved to SHT during the follow-up, this was observed either at the expense of only daytime BP, or at the expense of only nighttime BP, or at the expense of both daytime and nighttime BP. That was observed, respectively, in 11 (36%), 9 (24%), and 13 (40%) out of 33 subjects with NT and, respectively. in 28 (38%), 18 (29%), and 20 (33%) out of 66 subjects with UnWCHT.

Table 3 shows the annual evolution of BP during the follow-up in the three groups. In normotensives, the annual change of office BP tended to increase. In contrast, it decreased in both treated and untreated WCH but significantly more in treated WCHT than in untreated WCHT. Untreated WCHT showed a higher annual increase of 24 h, daytime, and NBP than untreated WCHT. Normotensives showed a lower annual increase of 24 h BP than untreated WCH but a higher annual increase of 24* *h BP than treated WCHT. During the follow-up, there were in the normotensives 16 (4.7%) cardiovascular events (10 strokes and 6 coronary episodes), in the untreated WCHT 28 (7%) events (18 strokes and 10 coronary episodes), and in treated WCHT 13 (8%) events (5 strokes and 8 coronary episodes), *p*=0.188, among all. All the events were nonfatal.

In the overall population and in all the subpopulations (NT, WCHT treated, and nontreated), there were no differences between individuals with or without transition to SHT regarding anthropometric (age, BMI, and gender) and biochemical parameters. However, in all populations, those with transition to SHT showed systematically significant higher values of systolic BP in office, 24 hours, daytime, and nighttime in comparison to those without transition to SHT. For example, in overall population, systolic BP values either in office, 24 hours, daytime, and nighttime were higher in subjects with transition to SHT (*n* = 122) vs. without transition to SHT (*n* = 767) i.e., respectively, 147 + 15 vs. 142 + 15, 123 + 5 vs. 121 + 6, 128 + 7 vs. 125 + 7, and 113 + 7 vs. 111 + 6 mm Hg (all *p* < 0.01).

## 4. Discussion

The aim of the present study was to evaluate the cumulative incidence of SHT in subjects with treated and untreated WCHT compared to normotensive subjects over 7.1 years of follow-up. Our main findings are that untreated subjects with WCHT defined by normalcy of both daytime and nighttime BP values show an increased risk of transition to SHT and an earlier progression to SHT in comparison to normotensive subjects. However, we cannot exclude that the higher age and baseline BP values of WCHT vs. NT may have influenced the greater propensity for the development of hypertension of WCHTs. At baseline, the SD of both 24 h SBP and 24 h DBP was significantly lower in the normotensive group as compared with the WCHT group which may also have contributed to the lower rate of progression of normotensives to sustained hypertension in comparison with WCHT. Treated WCHT appears to assume an intermediate position between untreated WCHT and NT thereby showing an annual evolution of BP values that were lower than both untreated WCHT and normotensives. In all populations (all together and in subgroups) those with a transition to SHT showed at baseline systematically significant higher values of systolic BP in office, 24 hours, daytime, and nighttime in comparison to those without transition to SHT. Thus we cannot exclude that higher baseline BP may represent an important determinant of the evolution to SHT. Another striking point of the study besides the classification of WCHT was the evaluation of 24-ABPM during two distinct moments of the follow-up that allowed us to establish a temporal evolution of the transition to SHT. We found that, at 30–60 months of the follow-up, the transition to SHT of untreated WCHT occurred earlier, being 2.7 times more frequent than normotensives. At the end of the follow-up, these values were about 1.7 times that of normotensives. Again, treated WCHT appears to assume an intermediate position between untreated WCHT and NT. The CV prognosis of WCHT and the risk of transition to SHT are still controversial. The alleged benignity of WCHT has been challenged and its long-term CV risk has been considered to be intermediate between NT and SHT [12, 14, 18, 27]. We hypothesised that an incomplete classification of individuals with WCHT may have contributed towards the conflicting data among the different studies. In fact, most of the related studies have used office BP (≥140/90 mm·Hg) and out-of-office BP (≤130–135/80–85 mm·Hg) thereby avoiding considering NBP values [6, 27–30]. In other words, nocturnal BP, which has been related strongly to the CV prognosis [8, 23, 24, 31, 32] was not considered in the WCHT definition in most of the available evidence, and this may have conditioned the results. We have recently found [8] in a follow-up of 7.4 years that WCHT patients who were classified on the basis of normal 24 h, daytime, and NBP showed a CV prognosis not different from NT but better than true hypertensives. This suggests that normal levels of NBP should be included in its classification to avoid misdiagnosis of WCHT, as suggested by some authors [33]. In general, a better prognosis of WCHT should be expected when normal NBP values are included in the definition of WCHT since NBP is a stronger predictor of risk than daytime BP [25]. In fact, a lower prevalence rate of WCHT [6, 34–36] and a lower value of left ventricular mass index [29] were found when normal values of daytime and nighttime BP values were considered together in the classification of WCHT compared with the condition of including daytime BP only. Moreover, the prevalence of WCHT was reduced when NBP was used for its categorization [36]. In the present study, as in our previous one [8], we have failed to demonstrate a clearly higher rate of events in the WCHT than in normotensives. However, because of the small number of events in differences between NT and WCHT populations a type 2 error cannot be ruled out. We found at the end of the follow-up a cumulative incidence of SHT in our WCHT populations between 10.5–16.5% which were significantly higher than in the normotensive population. In larger series, a 40% or higher propensity of WCHT to SHT was reported by others [20, 37, 38]. Some studies [39] found in WCHT of a greater prevalence of metabolic risk factors and a greater long-term risk of new-onset diabetes and a progression to sustained hypertension. In contrast, in our study at baseline anthropometric and biochemical characteristics and percentage of smokers of our WCHT population did not differ from that of normotensives which may also explain the relatively benign outcome and the lower percentage of transition to SHT of our WCHT population. Nevertheless, our data still support the prudent recommendations [1, 22, 32, 34] in relation to this entity of close surveillance with a frequent BP measurement outside the office. Since 24 h-ABPM enlightens the nighttime BP values, it is the preferential tool for the definition of WCHT unless new devices to home BP able to register the NBP will become available. There is also some debate whether drug therapy may influence the long-term evolution of BP and the CV outcomes of WCHT and whether or not patients with WCHT should receive antihypertensive therapy [7, 17, 36, 40, 41]. We were unable to contribute to clarify that dispute in view of the limited number of our TxWCHT subjects. To our knowledge, our study is one of the first studies that have prospectively studied WCHT classified on the basis of normal NBP and examined the long-term transition of to SHT using two moments of 24 h-ABPM after the baseline. Our study must be interpreted within the context of its strengths and potential limitations. First, the participants were selected from a regional database so that the results may not be extrapolated at a national or an international level. Second, because we were unable to evaluate all biochemical and anthropometric data during and at the end of the following, we cannot exclude that throughout that period changes in habits and behaviours may have influenced both office and ambulatory BP.

## 5. Conclusion

In our WCHT population selected by normalcy of daytime and NBP values, we found a faster and a significantly higher risk of transition from WCHT to SHT compared to normotensive subjects. Treated WCHT appears to assume an intermediate position between untreated WCHT and NT. Although we still lack robust evidence to confirm the benefit of antihypertensive treatment in the outcome of WCHT, the transition to SHT may be attenuated or delayed when antihypertensive therapy is undertaken. Although no increase in cardiovascular events was so far detected, these results support that WCHT subjects should be closely monitored for control of associated risk factors and early detection of progression to SHT, desirably with 24-ABPM.

## Figures and Tables

**Table 1:** Anthropometric biochemical data at baseline.

	NT (*n* = 434)	WCHT untreated (*n* = 399)	WCHT treated (*n* = 156)	*p* =
Age (years)	49 ± 14	51 ± 14^#^	51 ± 15^#^	**0.011**
Female (%)	52.9	51.1	52.4	0.665
BMI (Kg/m^2^)	27 ± 3	26 ± 4	26 ± 4	0.229
Smoking (%)	18	16	14	0.564
Glucose (mg/dl)	91 ± 022	97 ± 28	98 ± 25	0.171
Creatinine (mg/dl)	1.06 ± 0.27	0.99 ± 0.31	1.03 ± 0.40	0.783
Sodium (mEq/L)	139 ± 2	140 ± 3	140 ± 2	0.123
Potassium (mEq/L)	4.4 ± 0.8	4.3 ± 0.5	4.5 ± 0.10	0.160
Uric acid (mg/ml)	4.9 ± 1.4	5.2 ± 1.6	5.4 ± 1.7^*∗*^	**0.021**
Total cholesterol (mg/dl)	197 ± 35	199 ± 38	204 ± 39	0.259
HDL-C (mg/dl)	56 ± 14	56 ± 15	56 ± 16	0.864
LDL-C (mg/dl)	118 ± 33	119 ± 34	121 ± 34	0.320
Triglycerides (mg/dl)	117 ± 44	122 ± 49	121 ± 45	0.793
Albuminuria (mg/24* *h)	31 ± 17	32 ± 21	29 ± 20	0.546

NT, normotensives; WCHT, white-coat hypertensives; BMI, body mass index. Post hoc analysis: ^*∗*^*p* < 0.05 vs. NT and WCHT treated; ^#^*p* < 0.05 vs. NT.

**Table 2:** Blood pressure data.

	NT (*n* = 434)	WCHT untreated (*n* = 399)	WCHT treated (*n* = 156)	*p* *≤*
*In office-blood pressure and heart rate*				
SBPc baseline (mm Hg)	128 ± 8	151 ± 11^*∗*^	150 ± 10^*∗*^	0.001
DBPc baseline (mm Hg)	84 ± 9	91 ± 10^*∗*^	90 ± 10^*∗*^	0.001
HRc baseline (b/min)	78 ± 10	81 ± 9	80 ± 10	0.710
SBPc final (mm Hg)	131 ± 8.9	146 ± 11^*∗*^	143 ± 10^*∗*^	0.001
DBPc final (mm Hg)	86 ± 9	88 ± 10^*∗*^	86 ± 10^*∗*^	0.001
HRc final (b/min)	79 ± 11	80 ± 9	79 ± 11	0.261
Delta SBPc (mm hg)	3.0 ± 2.1	−3.9 ± 3.0^*∗*^	−6.1 ± 5.9^*∗*^	0.001
Delta DBPc (mm hg)	2.8 ± 3.7	−2.6 ± 2.7^*∗*^	−4.2 ± 2.1^*∗*^	0.001

*24 h-blood pressure and heart rate*				
SBP 24 h baseline (mm Hg)	120 ± 7	123 ± 5^*∗*^	122 ± 5^*∗*^	0.001
DBP 24 h baseline (mm Hg)	73 ± 7	75 ± 7^*∗*^	74 ± 7	0.001
HR 24 h baseline (b/min)	74 ± 9	73 ± 10	72 ± 10	0.159
SBP 24 h final (mm Hg)	122 ± 7	125 ± 6^*∗*^	123 ± 6^§^	0.001
DBP 24 h final (mm Hg)	74 ± 6	78 ± 7^*∗*^	73 ± 6^§^	0.001
HR 24 h final (b/min)	73 ± 8	72 ± 9	72 ± 9	0.311
Delta SBP24 h (mm Hg)	2.4 ± 3.1	2.9 ± 4.1^*∗*#^	0.7 ± 1.9^*∗*^^§^	0.001
Delta DBP24 h (mm Hg)	1.1 ± 2.4	1.2 ± 5.4^#^	0.23 ± 1.4^*∗*^^§^	0.001

*Daytime-blood pressure and heart rate*				
SBP daytime baseline (mm Hg)	124 ± 8	126 ± 6^*∗*^	126 ± 5^*∗*^	0.01
DBP daytime baseline (mm Hg)	77 ± 7	79 ± 7^*∗*^	78 ± 7	0.01
HR daytime baseline (b/min)	77 ± 10	76 ± 11	75 ± 10	0.170
SBP daytime final (mm Hg)	126 ± 8	129 ± 7^*∗*#^	127 ± 6	0.001
DBP daytime final (mm Hg)	78 ± 7	80 ± 7^*∗*#^	77 ± 7	0.01
HR daytime final (b/min)	76 ± 9	77 ± 10	74 ± 10	0.231
Delta SBP daytime (mm Hg)	2.2 ± 3.1	2.5 ± 6.0^*∗*#^	0.7 ± 2.8^*∗*^^§^	0.001
Delta DBP daytime (mm Hg)	1.0 ± 2.5	1.5 ± 6.1^*∗*#^	0.4 ± 1.8^*∗*^^§^	0.001

*Nighttime-blood pressure and heart rate*				
SBP nighttime baseline (mm Hg)	110 ± 8	111 ± 7	112 ± 8^*∗*^	0.01
DBP nighttime baseline (mm Hg)	65 ± 7	66 ± 7^*∗*^	66 ± 7^*∗*^	0.01
HR nighttime baseline (b/min)	66 ± 9	65 ± 9	66 ± 10	0.644
SBP nighttime final (mm Hg)	112 ± 9	114 ± 8^*∗*^	112 ± 8	0.013
DBP nighttime final (mm Hg)	66 ± 7	67 ± 8	65 ± 7	0.128
HR nighttime final (b/min)	67 ± 9	66 ± 9	67 ± 10	0.233
Delta SBP nighttime (mm Hg)	1.7 ± 3.0	2.1 ± 9.0^*∗*#^	0.5 ± 3.0^*∗*^^§^	0.001
Delta DBP nighttime (mm Hg)	0.6 ± 4.5	0.9 ± 6.9^*∗*#^	0.7 ± 2.8	0.01

*Nighttime BP fall (%)*				
Nighttime fall SBP baseline	10.7 ± 5.9	12.1 ± 6.5^*∗*^	12.7 ± 6.8^*∗*^	0.01
Nighttime fall DBP baseline	15.3 ± 7.4	15.9 ± 7.6	16.3 ± 7.6	0.259
Nighttime fall SBP final	10.3 ± 4.9	11.9 ± 5.8^*∗*^	12.0 ± 5.9^*∗*^	0.01
Nighttime fall DBP final	15.1 ± 6.5	15.3 ± 6.8	15.4 ± 6.8	0.232

NT, normotensives; WCHT, white-coat hypertensives; SBPc, office systolic BP; DBPc, office diastolic BP; HRc, office heart rate; Delta, difference between baseline and the end of the follow-up (final). Post hoc analysis: ^*∗*^*p* < 0.01 vs. NT; ^#^*p* < 0.01 different from WCHT treated; ^§^^*∗*^*p* < 0.05 WCHT treated vs. WCTH untreated.

**Table 3:** Annual evolution of BP (mm Hg/year) during the follow-up.

	NT (*n* = 434)	WCHT untreated (*n* = 399)	WCHT treated (*n* = 156)	ANOVA *p* <
SBPc (mm Hg)	1.2 ± 0.95	−1.36 ± 1.35^*∗*#^	−2.03 ± 1.56^*∗*^	0.01
DBPc (mm Hg)	1.01 ± 0.77	−0.98 ± 0.97^#^	−1.13 ± 0.75	0.01
SBP 24* *h (mm Hg)	0.53 ± 0.93	0.76 ± 1.06^*∗*#^	0.35 ± 1.01	0.01
DBP 24* *h (mm Hg)	0.22 ± 0.95	0.39 ± 1.11^*∗*#^	0.12 ± 0.71	0.01
SBP daytime (mm Hg)	0.54 ± 1.00	0.58 ± 1.48^#^	0.37 ± 1.34	0.01
DBP daytime (mm Hg)	0.28 ± 1.05	0.38 ± 1.94^#^	0.11 ± 0.92	0.01
SBP nighttime (mm Hg)	0.54 ± 0.84	0.63 ± 1.03^#^	0.33 ± 0.92	0.01
DBP nighttime (mm Hg)	0.21 ± 0.89	0.33 ± 1.00^#^	0.11 ± 0.87	0.01

NT, normotensives; WCHT, white-coat hypertensives; SBPc, office systolic BP; DBPc, office diastolic BP; HRc, office heart rate; Delta, difference between baseline and the end of the follow-up (final). Post hoc analysis: ^*∗*^*p* < 0.01 vs. NT ^#^*p* < 0.01 different from WCHT treated.

## Data Availability

Data are available in the Blood Pressure Unit Hospital Pedro Hispano, Matosinhos Portugal.
